# Cervical Osteophytosis Causing Tracheomalacia and Dyspnea

**DOI:** 10.7759/cureus.100995

**Published:** 2026-01-07

**Authors:** Laith Fada, Emma Karesh, John Wahidy, Shawn Clark

**Affiliations:** 1 Medicine, Alabama College of Osteopathic Medicine, Dothan, USA; 2 Research, Alabama College of Osteopathic Medicine, Dothan, USA; 3 Neurosurgery, Mobile Infirmary Medical Center, Mobile, USA

**Keywords:** chronic dyspnea, diffuse idiopathic skeletal hyperostosis (dish), osteophyte, osteophyte resection, tracheomalacia

## Abstract

Cervical osteophytosis, a common manifestation of degenerative spondylosis, can rarely exert mass effect to compromise adjacent airway structures. Acquired tracheomalacia secondary to anterior cervical osteophytes is exceptionally uncommon and results from dynamic airway collapse due to weakened tracheal cartilaginous support. Tracheomalacia may present with nonspecific respiratory symptoms such as dyspnea, cough, and orthopnea, and diagnosis is best established with direct visualization on bronchoscopy. We report a rare case of localized upper tracheal tracheomalacia caused by cervical diffuse idiopathic skeletal hyperostosis (DISH) with severe anterior osteophyte formation, resulting in clinically significant airway obstruction.

A 74-year-old male with a history of coal worker’s pneumoconiosis, osteoarthritis, DISH, and prior lumbar spine surgery presented with a six-year history of progressive dyspnea, recently complicated by audible resting stridor. He denied dysphagia or orthopnea. Bronchoscopy demonstrated dynamic upper tracheal collapse consistent with localized tracheomalacia. MRI and X-ray imaging revealed extensive anterior cervical osteophytes from C5-C7 producing marked posterior compression of the trachea and esophagus, accompanied by cervical spinal stenosis extending from C1-C6. Given progressive airway compromise, the patient underwent anterior cervical microsurgical osteophyte excision with posterior decompressive laminectomy and foraminotomies. Resection was confirmed intraoperatively with microscopy and fluoroscopy. Postoperatively, the patient exhibited resolution of stridor, improved respiratory function, and no new neurological deficits, and he remained symptom-free at follow-up.

This case highlights a rare but clinically significant cause of acquired tracheomalacia due to compressive cervical osteophytosis associated with DISH. Progressive dyspnea and stridor in patients with extensive anterior cervical osteophytes should prompt thorough airway evaluation, including bronchoscopy and cross-sectional imaging. Surgical decompression can provide durable symptomatic relief and restoration of airway patency in appropriately selected patients.

## Introduction

Cervical osteophytosis is a manifestation of degenerative spondylosis in which chronic spinal degeneration leads to the formation of bony outgrowths, or osteophytes, along the vertebral margins. While these osteophytes most commonly cause local pain or neurologic symptoms, they can rarely exert a mass effect - defined as direct physical compression - on adjacent structures, including the esophagus and airway. We present an unusual case of acquired tracheomalacia resulting from chronic compression of the trachea by a large anterior cervical osteophyte at the C3-C6 levels.

Tracheomalacia is a condition characterized by abnormal weakness of the tracheal cartilaginous framework, leading to excessive airway collapsibility during respiration. Depending on the underlying cause, tracheomalacia may be classified as diffuse or localized, with localized disease often resulting from prolonged external compression [1]. Common presenting symptoms are nonspecific and include cough, dyspnea, orthopnea, and, in more advanced cases, audible stridor. The patient in this case reported progressive dyspnea over six to seven years and was noted to have stridor on physical examination.

The pathophysiology of acquired tracheomalacia involves chronic mechanical stress on the trachea, which leads to cartilage weakening and laxity of the posterior tracheal wall, resulting in dynamic airway narrowing during expiration. One potential cause of such chronic compression is diffuse idiopathic skeletal hyperostosis (DISH), a noninflammatory condition characterized by excessive ossification of spinal ligaments and entheses, most commonly affecting the cervical spine. Bronchoscopy remains the diagnostic gold standard, as it allows direct visualization of dynamic airway collapse [2]. In this case, bronchoscopy demonstrated tracheomalacia localized to the upper trachea, while magnetic resonance imaging revealed severe cervical DISH with anterior ossification and spinal stenosis. The patient was subsequently referred to neurosurgery and underwent surgical excision of the offending osteophyte.

## Case presentation

A 74-year-old male with a history of coal worker’s pneumoconiosis, longstanding osteoarthritis, diffuse idiopathic skeletal hyperostosis, and previous lumbar spine surgery presented with a six-year history of progressive dyspnea, recently advancing to audible stridor at rest. He presented with worsening exertional limitations and dyspnea, but denied dysphagia, orthopnea, or other red-flag symptoms. He had a 40-year smoking history but quit in 2001.

**Figure 1:**
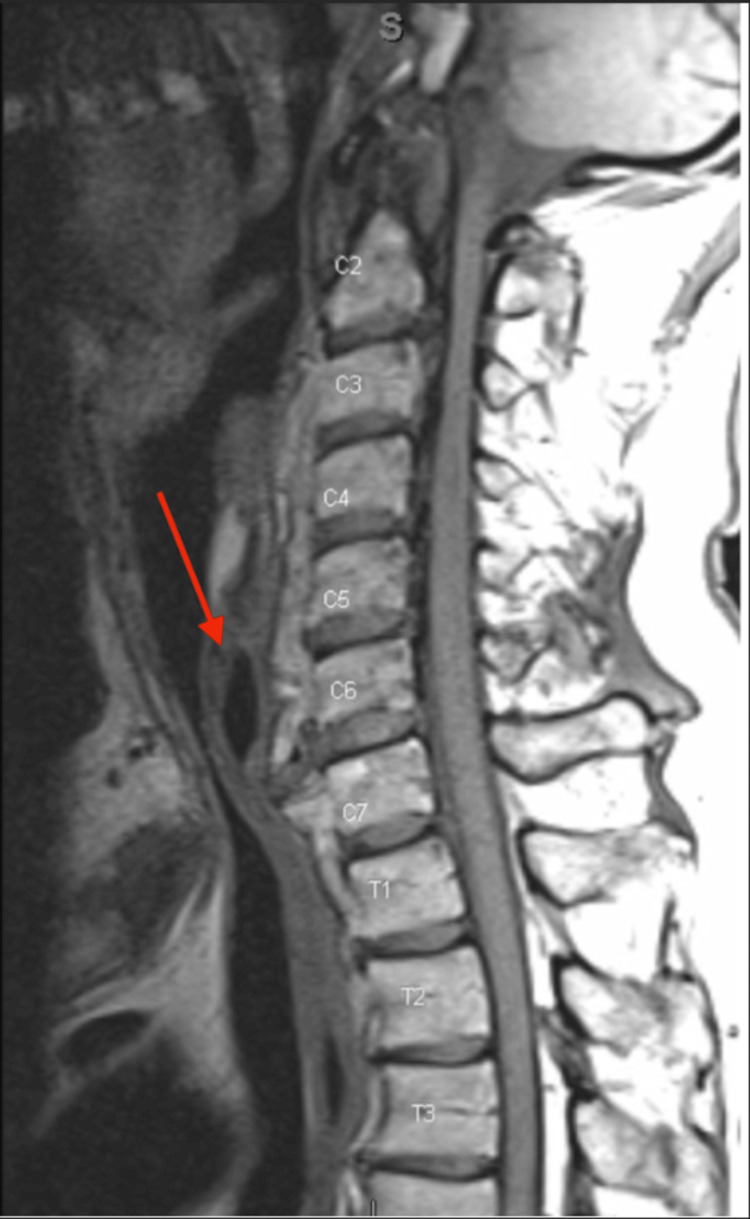
MRI of the cervical spine demonstrating extensive anterior osteophyte formation at C5–C7 (arrow).

**Figure 2:**
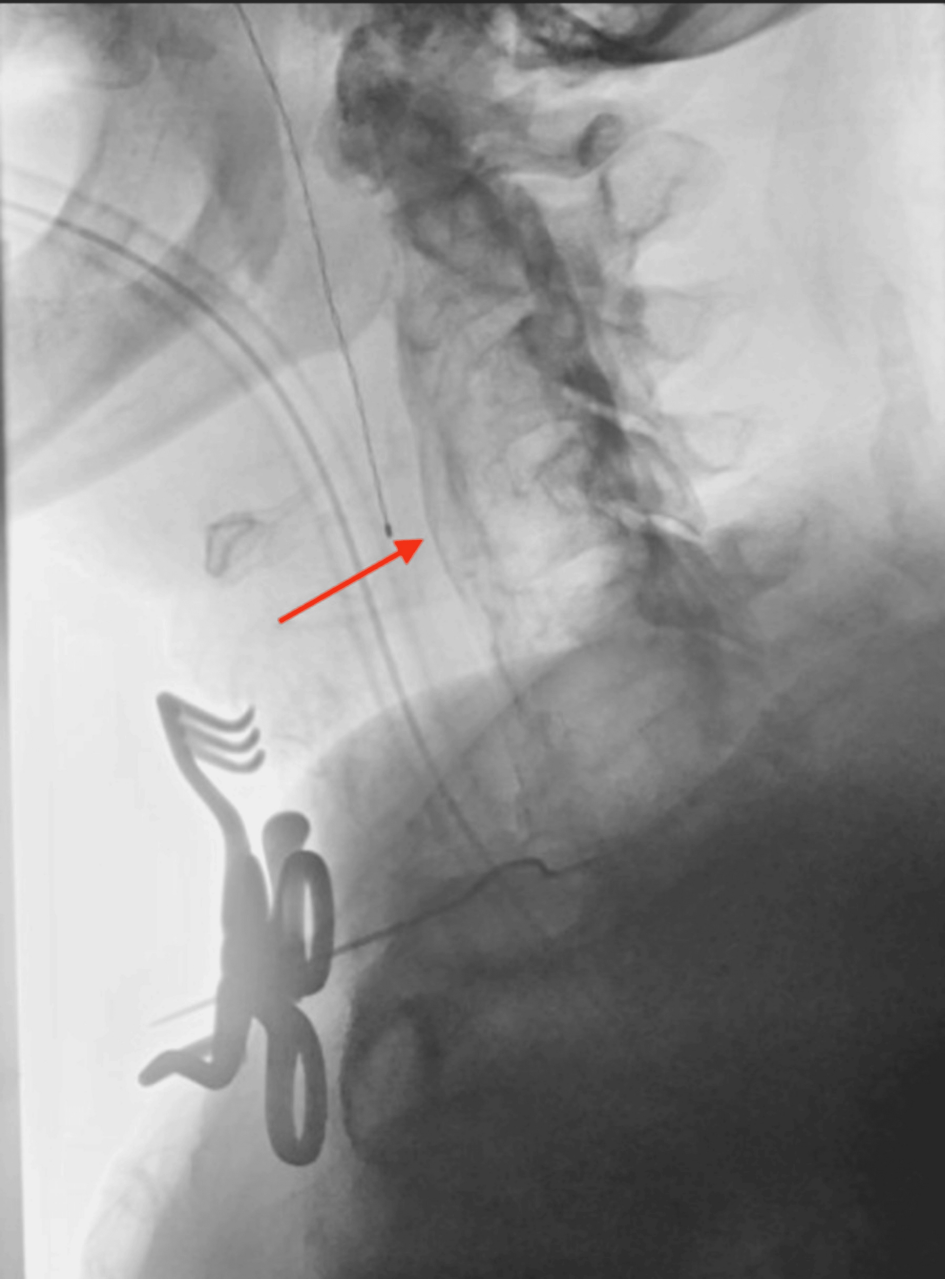
X-ray demonstrating causing posterior displacement of the trachea and esophagus (arrow).

Bronchoscopic evaluation revealed dynamic upper tracheal narrowing with collapse of the posterior tracheal wall consistent with localized tracheomalacia. Cross-sectional imaging, using MRI (Figure 1) and X-ray (Figure 2), demonstrated prominent anterior cervical osteophytes extending from C5 to C7, causing posterior tracheal and esophageal compression, as well as spinal stenosis from C1-C6.

Given the patient’s progressive airway symptoms and imaging findings, a multidisciplinary discussion concluded that surgical intervention was indicated to relieve airway obstruction and prevent further tracheal compromise. The patient underwent an anterior cervical approach with microsurgical removal of the C5-C7 anterior osteophytes, followed by posterior decompressive laminectomy (C1-C6) with partial facetectomies and foraminotomies for concurrent spinal stenosis. Intraoperative microscopy and fluoroscopy confirmed complete resection of the osteophytic mass.

Postoperatively, the patient demonstrated resolution of stridor and improved respiratory effort, with stable airway patency and no new neurological deficits. He participated in early physical therapy and was discharged home with outpatient rehabilitation. At follow-up, the patient remained free of airway compromise and reported significant improvement.

## Discussion

This case illustrates a rare presentation of tracheomalacia caused by cervical osteophytes due to Diffuse Idiopathic Skeletal Hyperostosis (DISH). While DISH can lead to ossification of spinal ligaments, airway obstruction and tracheomalacia are rare outcomes. Studies from 2011 showed that cervical osteophytes can lead to airway compromise via outward compression of the trachea [3]. Our patient's presentation shows noticeable C5-C7 osteophytes that likely led to chronic compression of the tracheal wall, leading to eventual tracheomalacia and dysphagia. This mechanism stresses the significance of dynamic airway evaluation via MRI imaging to explore the several possibilities of what may be causing the patient's symptoms.

Surgical management is the best way to treat airway obstruction from DISH when conservative measures fail. One report indicates that surgical excision of osteophytes along the anterior longitudinal ligament can help relieve dysphagia and respiratory distress [4]. The decision to perform an anterior cervical approach along with a posterior decompressive laminectomy led to the resolution of the airway compromise and spinal stenosis. This approach is not commonly found in literature, however, it presents the favorable outcome of resolving patients' symptoms via surgical intervention. 

Airway evaluation (via imaging or bronchoscopy) should be considered in all patients presenting with cervical DISH to explain possible dyspnea, dysphagia, or stridor that the patient may present with. Early recognition of airway involvement in these patients can prevent serious respiratory compromise in those who elect to undergo surgical intervention to resolve their symptoms. A multidisciplinary approach should be used in surgical planning, given the complex anatomic and physiologic dynamics that are involved.

## Conclusions

This case presents a rare example of tracheomalacia developing in the setting of diffuse idiopathic skeletal hyperostosis (DISH), highlighting the potential for chronic anterior cervical osteophyte compression to progressively weaken tracheal cartilaginous support. Such airway compromise may mimic more common pulmonary or cardiac pathologies, often leading to delayed diagnosis and prolonged morbidity. Early recognition of tracheomalacia in patients with advanced cervical osteophytosis is therefore critical, particularly when respiratory symptoms are unexplained or progressive. Prompt multidisciplinary evaluation and timely intervention can be lifesaving, prevent irreversible airway collapse, and significantly improve functional and respiratory outcomes.
